# Editorial: Advances in the treatment of acute severe ulcerative colitis

**DOI:** 10.3389/fgstr.2026.1924886

**Published:** 2026-07-22

**Authors:** Hirannya Karunadasa, Ronan O’Connor, Danny Con, Ashish Srinivasan, Abhinav Vasudevan

**Affiliations:** 1 Department of Gastroenterology, Eastern Health, Melbourne, VIC, Australia; 2 Department of Medicine, Austin Health, The University of Melbourne, Melbourne, VIC, Australia; 3 Eastern Health Clinical School, Monash University, Melbourne, VIC, Australia

**Keywords:** acute severe ulcerative colitis, inflammation, inflammatory bowel disease, therapeutics, ulcerative colitis

Acute severe ulcerative colitis (ASUC) is a severe manifestation of ulcerative colitis (UC). Over time, the management of ASUC has changed from largely supportive care, historically associated with high mortality rates, to structured inpatient algorithms allowing timely use of rescue medical therapy and consideration of colectomy when indicated. These advances, together with improvements in multidisciplinary care, have reduced ASUC related mortality to less than 1%. However, in patients with ASUC, colectomy rates remain a major unresolved outcome, with 20% to 40% of patients requiring colectomy after one or more episodes of ASUC hospitalisations (1).

Current ASUC management pathways rely on rapid identification of at-risk patients, exclusion of enteric infection, early commencement of intravenous corticosteroids, daily objective reassessment and prompt escalation to rescue therapy or colectomy in patients failing to respond (2). Infliximab, an anti-tumour necrosis factor (TNF) monoclonal antibody, is widely used as first-line rescue therapy for patients failing to respond to intravenous corticosteroid therapy and is well supported in both randomised controlled and large observational studies, although calcineurin inhibitors remain an established alternative in selected patients (3, 4). However, the effectiveness of infliximab is closely linked to adequate drug exposure, with lower day 3 serum infliximab levels and higher early infliximab clearance predicting treatment failure and colectomy in ASUC (5). Unlike milder manifestations of UC, multiple factors including high inflammatory burden and upregulation of TNF causing more rapid utilisation of infliximab, elevation of proteolytic clearance and drug loss through the highly permeable inflamed intestinal mucosa, influence infliximab levels in ASUC (6).

Another major challenge in the management of ASUC is early identification of patients unlikely to respond to first line advanced medical therapy. Historical indices, including the Travis criteria (7), were developed to predict corticosteroid failure and guide consideration of colectomy, with more recent use to also determine escalation to medical rescue therapy. However, with increasing use of advanced therapies, there remains a need for validated tools that can predict failure of rescue medical therapy and guide personalised treatment decisions. In this context, Karthikeyan et al. highlight ongoing efforts to optimise infliximab use, including pharmacokinetic modelling and development of dosing dashboards, such as those being explored by the TITRATE trial investigators, to individualise therapy according to patient and disease-related factors including weight and serum albumin (8, 9).

The emergence of Janus kinase (JAK) inhibitors represents an important development in the management of moderate to severe UC and potentially ASUC. JAK inhibitors block activation of JAK-signal transducer and activator of transcription (STAT) pathway, a key intracellular signalling pathway through which multiple cytokines mediate and propagate intestinal inflammation. Karthikeyan et al. discuss the potential role of JAK inhibitors in the management of ASUC and propose their incorporation into clinical algorithms as a salvage therapy following failure of infliximab rescue therapy. Although ASUC-specific randomised controlled data remains limited for JAK inhibitors, their rapid onset of action and broad cytokine inhibition make them attractive candidates for further evaluation in this setting.

Similarly, interleukin (IL)-23 p19 inhibition represents a novel but more selective anti-inflammatory strategy in IBD. IL-23 contributes to the maintenance of Th17-mediated inflammation, which is implicated in the pathogenesis of UC and Crohn’s disease. In the systematic review and meta-analysis by Wang et al. demonstrate that IL-23 p19 inhibitors, including risankizumab, guselkumab and mirikizumab, are effective for induction and maintenance of clinical, endoscopic and histological remission in IBD compared with placebo, with a favourable safety profile. While these agents are not yet established in ASUC, they represent an important expansion of the therapeutic armamentarium and may inform future treatment strategies for severe disease phenotypes.

Beyond direct suppression of inflammatory mediators, restoration and maintenance of the intestinal epithelial barrier has been increasingly recognised as a potential therapeutic target in IBD. Mao et al. demonstrate that rhynochophylline, a bioactive alkaloid derived from Uncaria rhynochophylla, may ameliorate inflammatory colitis in murine models through modulation of the aryl hydrocarbon receptor (AhR)-nuclear receptor subfamily four group A member 1(NR4A1) pathway and upregulation of key tight junction proteins. Similarly, Liu et al. identify the hepatocyte nuclear factor 1β (HNF-1β)/solute carrier family 26 member 3 (SLC26A3) pathway as a potential therapeutic axis and show that p-hydroxy benzaldehyde, a phenolic compound derived from Nostoc commune, may regulate tight junction and adherens junction proteins in murine colitis models. Although they are preclinical, these studies highlight the importance of intestinal epithelial barrier integrity and the potential for therapeutic strategies that move beyond broad immunosuppression which may be useful in future as adjunctive or complementary agents in IBD.

Repurposed therapies may also provide complementary approaches in UC. In a small, randomised pilot trial of mild to moderate UC patients conducted by Khrieba et al., add on high dose atorvastatin therapy to 5-aminosalicylates demonstrated improvement in clinical measures, quality of life domains and inflammatory markers over placebo. Although such an approach is unlikely to have immediate role in ASUC, this study illustrates the value of investigating utility of widely available therapies which may have potential immunometabolic or anti-inflammatory effects, particularly in the context of an ageing IBD population where therapies could be used for multiple indications.

Despite major improvements in ASUC care, improving colectomy-free survival remains a critical unmet need. Future progress will require a deeper understanding of the pathophysiological processes that drive severe colonic inflammation, validated risk stratification tools to distinguish low-risk from high-risk disease, and optimised treatment algorithms that define the appropriate dosing, sequencing and combination of advanced therapies. The studies included in this Research Topic, collectively highlight the evolving therapeutic landscape of UC and ASUC, spanning pharmacokinetic optimisation of established rescue therapies, novel targeted immunomodulation, epithelial barrier restoration and therapeutic repurposing. Together, their reinforce the need for rigorous translational and clinical research to improve outcomes for patients with severe UC.
